# Biophysical studies of modified PVC sheet based on sunflower oil for antistatic and blood bags applications

**DOI:** 10.1038/s41598-024-62709-5

**Published:** 2024-06-06

**Authors:** Shimaa Farag Hamieda, Abeer Reffaee, Mona Saied

**Affiliations:** Microwave Physics and Dielectrics Department, National Research Centre, Giza, Egypt

**Keywords:** PVC sheet, SSO, Dielectrics, Surface roughness, Hemocompatibility, Biophysics, Physics

## Abstract

In this work, the surface of polyvinyl chloride PVC sheet was modified by blending it with sunflower seed oil SSO to obtain PVC sheet/SSO films of ratios 100/0, 90/10, 80/20, 70/30, 60/40, and 50/50 (v/v)% using the solution casting method. Various techniques were used to characterize the prepared films, besides the use of hemolysis assays and blood clot formation tests. FTIR spectra revealed that there was a good interaction between the PVC sheet and the oil. The dielectric measurement indicated that SSO addition enhanced the dielectric properties of the sheet. The study of dielectric relaxation times confirmed the interaction between SSO and the sheet. DC conductivity increased to 6 × 10^–6^ S/m, so it could be applied in antistatic applications. Also, SSO addition increased the value of the thermal stability. According to SEM micrographs, the film was roughened at a ratio of 60/40 and smoothed out at 50/50. This behavior was confirmed with roughness and contact angle measurement results, in which the film of ratio 60/40 had the highest value equal to (72.03°) and then decreased at 50/50 to (59.62°). These results were confirmed by XRD measurement as the crystallinity increased at the film ratio of 60/40 and decreased again at 50/50. Also, the ratio of 60/40 demonstrated a large decrease in thrombus weights along with a slight increase in hemolysis, which is within the acceptable range and has a high degree of biocompatibility, so this concentration is recommended to be used in blood bags applications.

## Introduction

Synthetic polymers have attracted a lot of attention in the past decade for both technical and medical uses because of their wide range of physical and chemical properties^1^. Several synthetic, biological, and hybrid polymers have been used in a variety of medicinal applications. There is a large variety of polymers available, and they additionally have the benefit of having customizable physical, chemical, and biological properties to meet the requirements of certain applications^2^. Polymer dielectric characteristics can be measured as a function of electric field frequency using dielectric spectroscopy technique. More details regarding the intermolecular activity inside the polymer or polymer composites may be revealed by the resultant spectrum^3^.

Polymeric materials have already been used in blood-contact devices such as therapeutic embolization microparticles, drug delivery nanoparticles, large vascular grafts, artificial hearts, oxygenators, and renal dialyzers^1,4^. All of these are intrinsically incompatible with blood, and they cause a variety of reactions. Polymeric membranes need to be modified using a variety of methods to increase their biocompatibility^4^.

Among commodity polymers, polyvinyl chloride (PVC) is the most versatile. It can meet a variety of requirements for cost, performance, safety, and product function. Due to its biological activity, low cost, workability, and ease of manufacturing and manipulation, PVC has been the most widely used plastic in a wide range of applications in everyday life^5^. PVC alone has restricted usage; to create a plastic that is useful and has a wide range of qualities, it must be combined with other additives that improve the flexibility, softness, antistatic properties of PVC^6^.

A variety of plasticizers are used in improved PVC processing technology. PVC is made soft or flexible by adding plasticizers in the appropriate amounts to achieve the desired degree of elasticity. Because of its exceptional capacity to produce large amounts of plasticizer, PVC is among the most versatile polymers^7^. Many medical products include plasticized PVC-based film, sheets, and tubing. The majority related to applications that come into contact with blood^8,9^.

Whereas surface modification mainly affects surface properties, the PVC formulation determines the properties of both bulk and surface. Attention is placed on the connections between blood compatibility, PVC surface modification, and chemical formulation. The PVC formulation, particularly with regard to plasticizer selection and level of incorporation, was also found to have a significant impact on plasticizer surface dispersion and blood compatibility^10,11^. Also, among the various qualities of materials, surface roughness is a common and important characteristic^12^. For thin layers or films, surface topography is important to their mechanical, physical, and dielectric properties^13^. It is well recognized that surface shape, stiffness, wettability, and roughness influence the adhesion of various cell types^14^.

The composition of the blood and the surface, which is determined by its physicochemical characteristics such as surface area, crystallinity, hydrophobicity/hydrophilicity, roughness, flexibility, and electrical properties, determines how blood and a surface interact^15^. Moreover, blood clots occur as a result of blood and medical device surfaces interacting. Medical device innovation is still plagued by the frustrating problem of device-induced thrombus development. Hemolysis, embolization, and device malfunction can all be caused by the presence of clots. Therefore, it is necessary to prevent or reduce the processes of surface adsorption, adhesion, and activation of thrombus formation^16^. With respect to the formulation, plasticizer selection is critical in the medical application of PVC.

Conductive polymers offer great electrical and optical properties, a high conductivity/weight ratio, and the ability to be made porous and biodegradable. They have also been used, improving the biocompatibility. Plasticizers derived from vegetable oils are environmentally benign plasticizers named biochemical plasticizers^17^. They may acquire improved properties and replace synthetic primary plasticizers. Antistatic agents are artificial additives that are applied to surfaces or materials to prevent the accumulation of static electricity. Materials known as anti-statics work to prevent static electricity from building, especially on polymer surfaces. Due to the charge buildup at the surface, the material is prone to static cling, dust adhesion, and electric discharges^18^. Despite the foregoing, concerns about the impact of common synthetic plasticizer and antistatic agent exposure on the environment and human health are increasing^19^. Research on bio-based additives is becoming more and more popular as a way to reduce the polymer industry's dependency on petrochemical feedstocks^20^. Vegetable oils that originate from inedible sources have drawn considerable attention due to their easy availability, low toxicity, and intrinsic biodegradability^19^. So, the main goal of our research is to prepare echo-friendly films by blending and modifying the PVC sheet with sunflower oil as a bio plasticizer and bioantistatic agent in different ratios in order to improve their biophysical properties for blood bags or antistatic applications.

## Experimental

### Materials and methods

#### Materials

Polyvinyl chloride sheets were obtained from the European Council of Vinyl Manufacturers, a division of the Association of Plastics Manufacturers in Europe.

Sunflower oil (SSO) was produced from ground sunflower seeds at oil mills in the Ismailia region of Egypt. Tetrahydrofuran of molecular weight (72.11) was purchased from Sigma Aldrich. Formaldehyde 36% from ALFA Chemical. Ca Cl_2_, molecular weight (110.98) from Sigma Aldrich.

The sunflower seed used in this research is the main source of sunflower seed oil all over the world and presents a large amount. This research Involves species not at risk of extinction and complies with relevant institutional, national, and international guidelines and legislation.

#### Methods

##### Preparation of PVC Sheet/SSO films

PVC sheet/SSO films of ratios (100/0, 90/10, 80/20, 70/30, 60/40, and 50/50) v/v% were prepared by the solvent casting technique using tetrahydrofuran as a solvent. Solutions with a total polymer and SSO concentration of 5 wt% were prepared. The volume of each component solution, PVC sheet and SSO, was calculated to obtain films of 0.3 mm thickness. These components were mixed together for one hour under magnetic stirring. The mixed solutions were then poured into Petri dishes, and the solvent was slowly evaporated under ambient conditions. The resulting films were dried at 37 °C for 48 h. The thickness of the films was measured by micrometers^21^.

##### Attenuated total reflection- fourier transform infrared spectroscopy (ATR -FTIR)

Using the JASCO FT/IR 300 E (Tokyo, Japan), the functional groups in the PVC sheet /SSO film backbones were identified and recorded. 400–4000 cm^−1^ measuring range using ATR-FTIR spectroscopy**.**

##### Scanning electron microscope (SEM)

A scanning electron microscope was used to analyze the surface morphology, bulk, and structural alterations of the produced films. Electron micrographs have been obtained using energy dispersive spectroscopy (EDX) equipment equipped with a scanning electron microscope (SEM) of the Philips XL30 Japan type.

##### X-Ray diffraction (XRD)

The amorphous or ordered nature of the prepared samples was examined by diffractometer (Bruker AXS D8 Advance) using Cu Kα X-ray tube radiation (λ = 1.5406 A) with a scanning mode detector and a Gobel mirror. Measurements were made over the range of 4° to 70° on a 2θ scale with 0.4 s as a dwell time.

##### Dielectric measurements

The dielectric measurements and conductivity of the samples were obtained using a high-resolution broadband impedance analyzer (Schlumberger Solartron 1260). The frequency range of the applied AC electric field was 0.1 Hz to 1 MHz. Both the measurements and the calculations were done automatically. A GPIB IEE488 cable was used to connect the impedance analyzer to a personal computer. Data was obtained using the commercial interfacing and automation program LabVIEW. There is more information available elsewhere^22^. Permittivity ε′ and dielectric loss ε" have error rates of 1% and 3%, respectively.

##### The differential scanning calorimetry (DSC)

DSC analysis was carried out in the Central Laboratories Network, National Research Centre (NRC), Egypt, using a DSC131 evo differential scanning calorimeter (SETARAM Inc., France). The standards (mercury, indium, tin, lead, zinc, and aluminum) were used to calibrate the device. The purge gases that were employed were nitrogen and helium. The test was designed with a heating zone that ranged from 20 °C to 100 °C with a heating rate of 10 °C per minute. The samples were placed in the DSC after being weighed in an aluminum crucible (30 ul). Calisto Data Processing Software Version 149 was utilized to process the thermogram results.

##### Thermogravimetric analysis (TGA)

The Perkin-Elmer TGA 7 (USA) device was used to measure the samples' thermal stability. In a nitrogen atmosphere, the heating rate was 10 °C/min up to 700 °C.

##### Optical properties using ultraviolet–visible spectroscopy

Each film was cut into a 4.5 × 0.5 cm rectangle sample, which was inserted into a quartz cell and put into a Cintra 303 UV–Vis spectrophotometer (made by GBC Scientific Equipment, located in Mexico City). The reference utilized was air. Every film was tested three times, and spectra were obtained at wavelengths ranging from 200 to 1600 nm. The results were reported using averages of the transmittance percentage (T%). The optical energy band gap (Eg) and refractive index (n) are calculated using the following equations:

$$\alpha \, = \,{2}.{3}0{\text{3 A}}/{\text{t}}.$$

(hυ)^2^ = *B*(hυ−E**g**) for the direct transition Where hυ is the photon energy, αis the absorption coefficient, A is the absorbance, t is the film thickness and B is a constant.

##### Surface roughness measurement

The surface roughness of the samples was characterized by using AFM coupled with a WiTec Alpha 300R Raman Imagining Microscope. The AFM images were measured in AC mode.

##### Contact angle measurement

To evaluate wettability, a contact angle tester was used to assess the films. It used distilled water as the testing liquid. The surface was cleared of dust particles using nitrogen. The sessile drop method was used to assess static contact angles. A minimum of 5 drops were used per sample. Both sides of the film were analyzed. For every sample, five measurements were made, and average values were determined.

##### Hemocompatibility studies

The prepared PVC sheet/ SSO films, with and without SSO, were submitted to in vitro studies for the preliminary evaluation of hemocompatibility.

##### Hemolysis assay

The hemolysis assay was performed as mentioned by (19), A blood/water mixture was used as the positive reference, and a blood/saline mixture served as the negative control. The percentage of hemolysis was calculated using the following equation:

$${\text{Hemolysis }}\% \, = \,\left[ {\left( {{\text{A}}_{{{\text{sample}}}} - {\text{ A}} -_{{{\text{control}}}} } \right)\left] { \, / \, } \right[\left( {{\text{A}}_{{ + {\text{ control}}}} - {\text{A}}_{{ - {\text{ control}}}} } \right)} \right]\, \times \,{1}00.$$

Where A is the absorbance.

##### Blood clot formation test and blood clot index

Anti-thrombogenic potential, judged by a blood clot formation test, was performed as mentioned by Refs.^23,24^. In this test, the films were cleaned at 37 °C for 24 h using saline water (0.9% w/v NaCl). To initiate the thrombus formation, 0.3 ml of citrated blood and 0.02 ml of CaCl_2_ solution (4 mol/L) were added to these films. 4.0 ml of distilled water was added to stop the reaction, and the resulting thrombus was separated by soaking in water at room temperature for ten minutes. After that, the clot was fixed for another ten minutes in a 36% formaldehyde solution (2.0 ml). The fixed clot was placed in water for 10 min, and, after drying, its weight was recorded. The same procedure was repeated for the glass surface as a control, and the respective weights of the thrombus formed were recorded. In order to calculate the blood clot index, the absorbance of free hemoglobin in distilled water was measured at 540 nm. The control for 100% free hemoglobin was obtained from blood on a glass surface. The percentage of BCI was calculated using the following equation:

$${\text{BCI }}\% \, = \, \left[ {\left( {{\text{A}}_{{{\text{sample}}}} /{\text{A}}_{{{\text{control}}}} } \right)} \right] \, \times {1}00,$$

where A is the absorbance.

### Ethical approval

All the methods were carried out in accordance with relevant institutional guidelines and regulations.

## Results and discussion

### Fourier transform infrared spectroscopy (ATR -FTIR)

**Figure 1:**
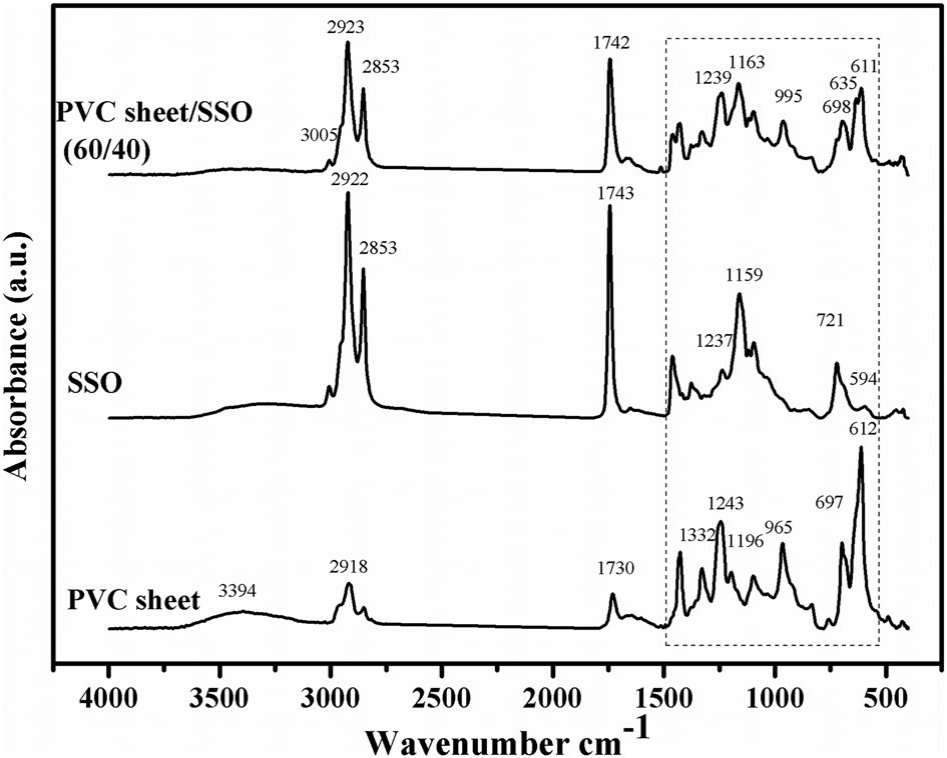
FTIR spectra of PVC sheet, SSO and PVC sheet /SSO (60/40).

The interaction between SSO and PVC sheet was studied by FTIR microscopy. FTIR spectra for PVC sheet, SSO, and PVC sheet /SSO (60/40) have been plotted in Fig. 1. The stretching vibration of the hydroxyl (OH) group has a characteristic peak at 3000–3600 cm^−1^, while that at 3005 cm^−1^, in the SSO spectrum, is attributed to the carboxylic OH stretch molecule, which also appeared in the PVC sheet/SSO (60/40) blend, indicating a good interaction between the oil and the polymer, and at 2926–2853 cm^−1^ for C–H stretching vibrations that are symmetric and asymmetric, respectively^25^. The characteristic band for the ester carbonyl functional group of the triglycerides appeared at 1730 cm^−1^ in the PVC sheet, overlapped with the 1742 cm^−1^ band in SSO, and appeared at 1741 cm^−1^ band in the blend film. The band at 1332 cm^−1^ in PVC sheet is related to CH bending, while the band at SSO is attributed to CH_2_CH bending. The band at 1243 cm^−1^ in the PVC sheet and 1239 cm^−1^ in SSO appeared at 1237 cm^−1^ in the blend, which is ascribed to the C–C swing vibration in the group CH_2_–CHCl. Also, 1196 cm^−1^ in PVC sheet and 1159 cm^−1^ in SSO overlap at 1163 cm^−1^ in the blend, which is attributed to C-O stretching, indicating a good interaction between the oil and the polymer matrix. The peak existing at 1097 cm^−1^ in both spectra of SSO and PVC sheet is for the C=O stretch. The peaks at 965 and 913 cm^−1^ are due to the =C–H bend appearing at 963 cm^−1^ in the blend, and the peaks at 834 and 848 cm^−1^ overlapped at 836 cm^−1^ are for =C–H. The bands at 758 cm^−1^ in PVC sheet and 721 cm^−1^ in SSO overlapped with the band at 697 cm^−1^ and appeared as a broader band at 698 cm^–1^. This indicates a good interaction between the oil and the PVC sheet. The absorption related to C–Cl stretch is found at 697 and 612 cm^–1^ in PVC sheet with higher intensity, which decreased greatly in the spectrum of 60/40 film, indicating a good interaction between Cl atoms in the polymer and the oil^26,27^.

### Scanning electron microscope (SEM)

**Figure 2:**
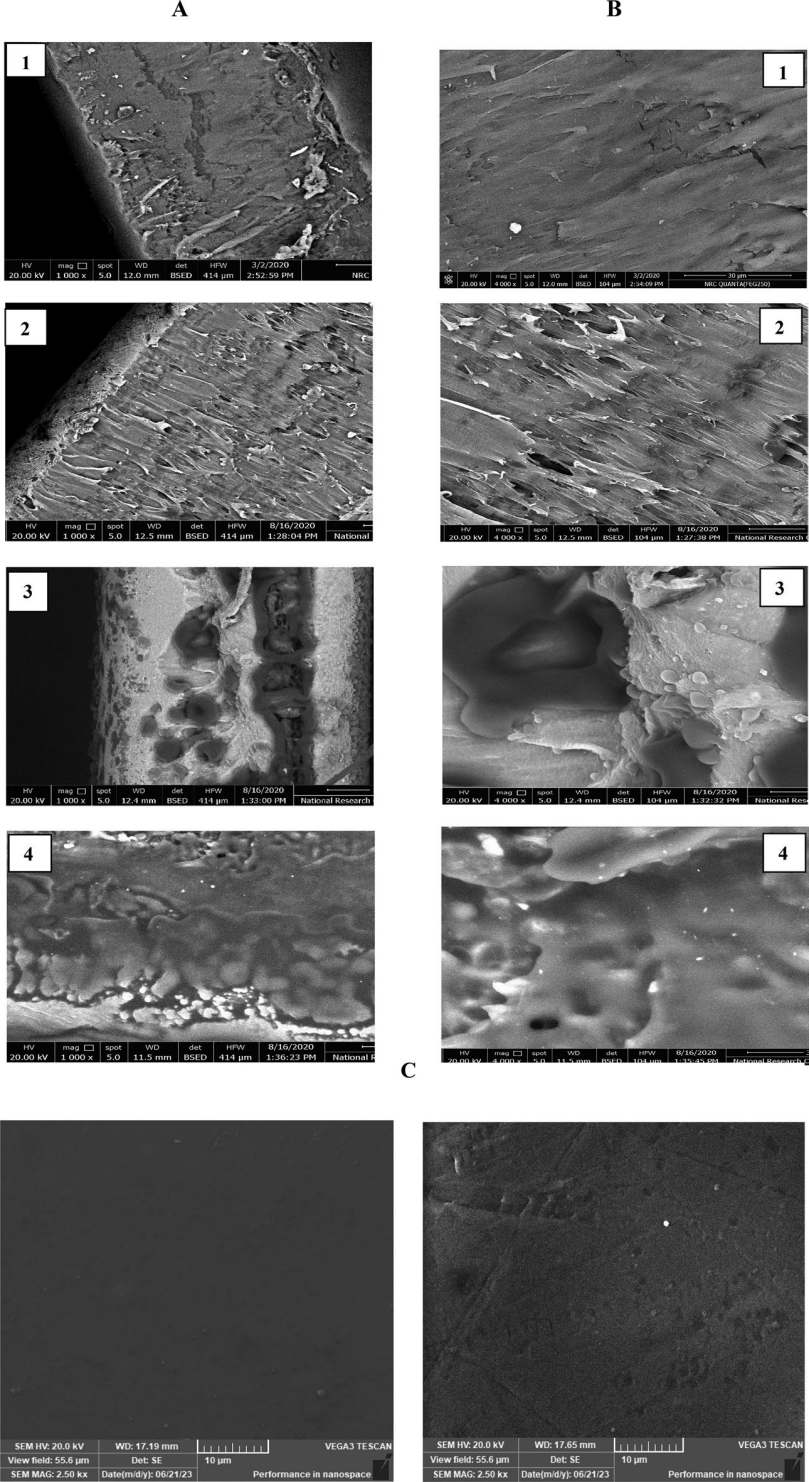
SEM micrographs (1000 x) (**A**) (1, 2, 3 & 4) and (4000 x) (**B**) (1, 2, 3 & 4) of fractured surfaces for PVC sheet /SSO (100/0, 90/10, 60/40 &50/50) v/v% and (**C**) (a and b) SEM surfaces for (100/0 &60/40) respectively.

SEM micrographs (1000 and 4000 ×) of fractured surfaces for PVC sheet / SSO of ratios (100/0, 90/10, 60/40 and 50/50) v/v% were illustrated in Fig. 2A (1, 2, 3 and 4) and B (1, 2, 3 and 4) respectively, and micrographs of the surface for (100/0, and 60/40) were illustrated in Fig. 2C(a and b). It is obvious from the figures that the surface of the control PVC sheet (100/0), Fig. 2A1 and B1, is homogenous and smooth, and there was good interference between the PVC sheet and SSO by introducing SSO into the film for 90/10, 60/40, and 50/50 blends. Furthermore, there are no fractures, and it is noticeable for the film with a blend ratio of 60/40, (Fig. 2A3 and B3). By increasing the SSO concentration, the film is not fully saturated with the oil, and the oil interference makes the surface less smooth and becomes rough. While for the 50/50, the film became fully saturated with the oil, the surface became smoother and more homogenous, and the surface roughness decreased.

### X-ray diffraction (XRD)

**Figure 3:**
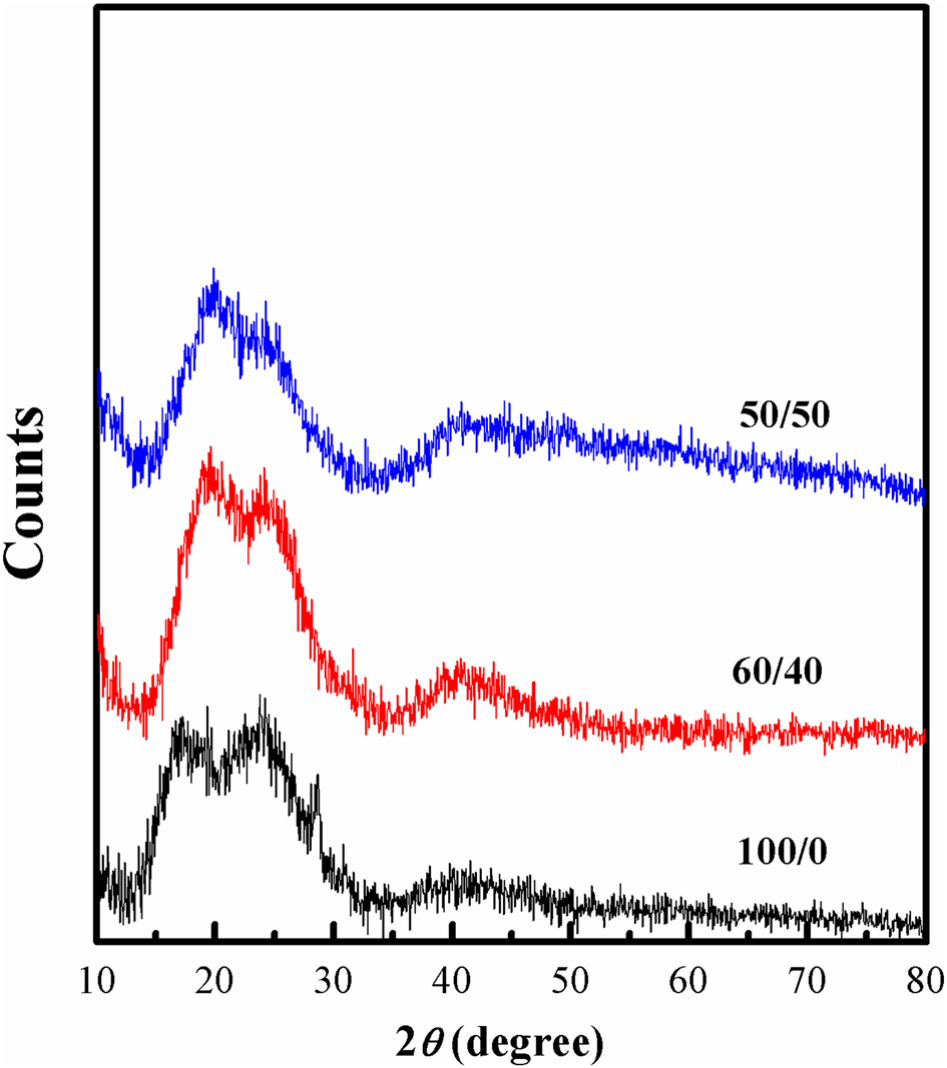
XRD pattern of PVC sheet/SSO films of ratios (100/0, 60/40 and 50/50).

XRD pattern of the prepared films of ratios (100/0, 60/40 and 50/50) is shown in Fig. 3. From the figure, we note that the PVC sheet and its blends with SSO have broad spectra and there is no diffraction sharp peak observed and the samples are amorphous in nature. The PVC sheet has a broad peak at 2Ө = 19.2 and its intensity increased by blinding with SSO and the broad peak became sharper as seen in the figure for the film of ratio 60/40. This means the crystallinity of the film increased at this concentration. By increasing the oil concentration, for 50/50 film, the broad peak sharpness and intensity decreased again and its width decreased. This decrease in the peak intensity is related to disorder in PVC structure^21^.

### Dielectric properties

**Figure 4:**
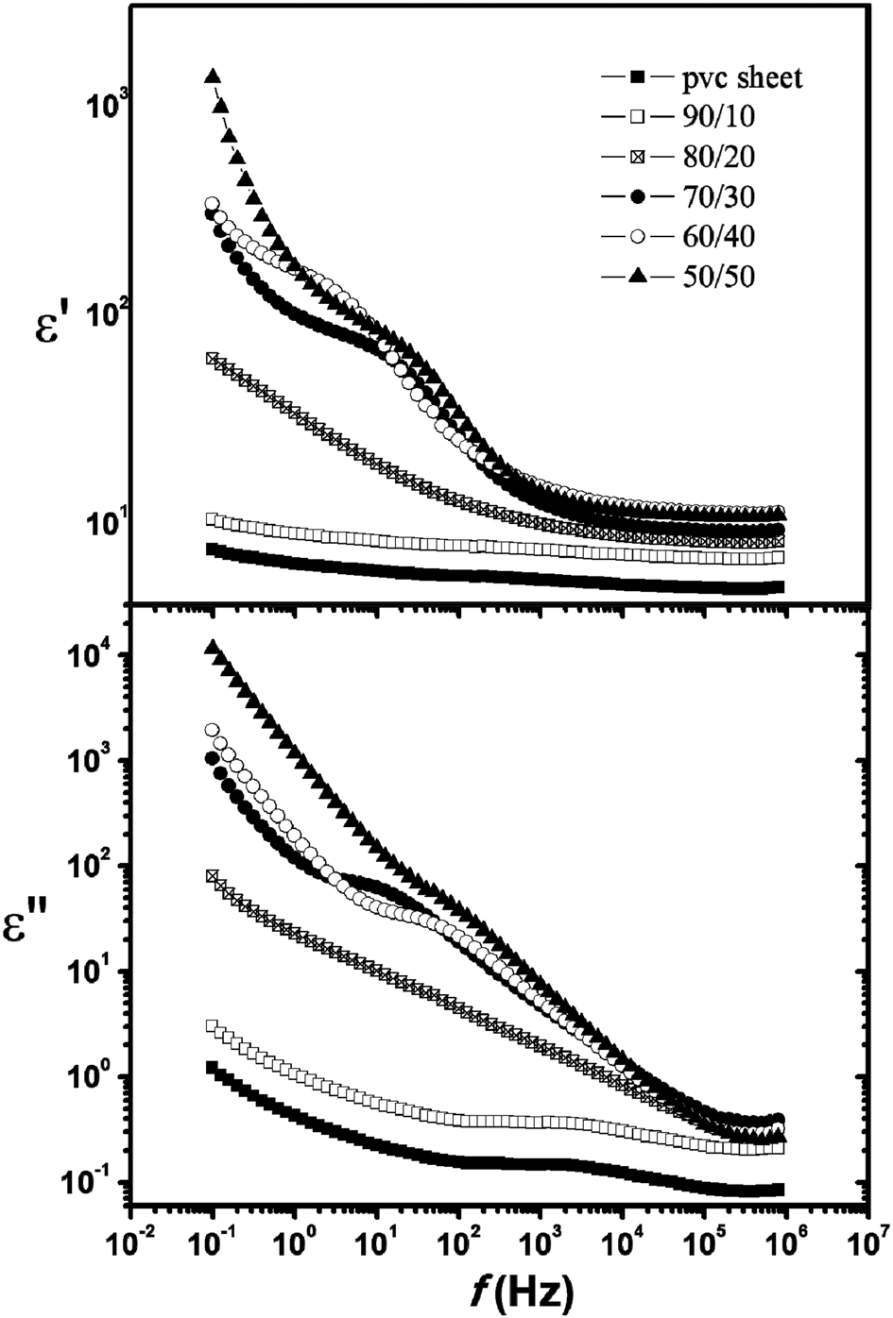
The permittivity ε′ and dielectric loss factor ε" of PVC sheet /SSO films of blend ratios (100/0, 90/10, 80/20, 70/30, 60/40 and 50/50) v/v% at 30 °C.

One effective technique for studying the molecular interactions in polymer composites is dielectric analysis. Figure 4a displays the frequency dependence of dielectric loss (ε") and permittivity (ε ') of PVC sheet and its blends with SSO at room temperature. It is evident that as the frequency increases, the values of ε' fall because of a decrease in dipoles contributing to polarization. As the SSO content increases, the permittivity (ε') of the PVC sheet increases, particularly at lower frequencies. The reason for this phenomenon is interfacial polarization, which is often caused by low-frequency dispersion and results from the composites’ heterogeneity^28^. Roughness in thin dielectric films causes non-conforming contact between the electrodes and the samples under investigation, resulting in air gaps at the interfaces^13^. Since air has the lowest dielectric constant (ε ' = 1)^29^, air gaps caused by roughness have a negative effect on the dielectric constant of investigated films. In Fig. 4a, the drop in the dielectric constant for the 60/40 PVC/SSO blend is explained by an increment in roughness. The roughness measurements showed that the 60/40 PVC/SSO blend has the highest roughness. It is evident from Fig. 4b that there is more than one relaxation process occurring since the curves pertaining to ε" and log f are broader than the Debye curve.

**Figure 5:**
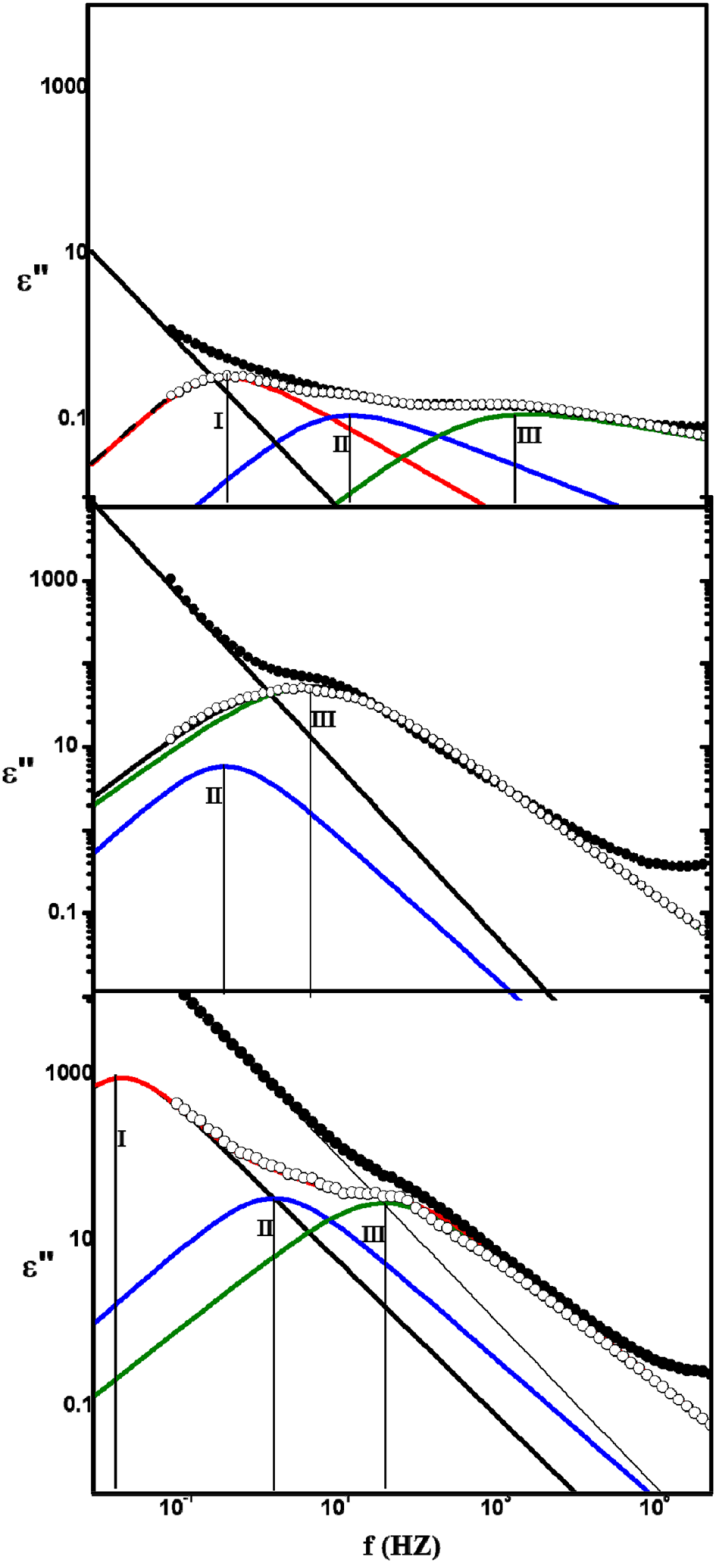
The analyses curves for PVC sheet /SSO films of ratios (100/0, 60/40 and 50/50) v/v%.

A computer program was used to fit the data. A superposition of three Havriliak-Negami functions provided the best fit to the data, in addition to the conductivity function. Fitting of ε" data for 100/0, 60/40, and 50/50 blends is illustrated in Fig. 5 as examples for analyses. As seen in the figure, the mobility of space charges deposited on the polymeric sample near the electrode is the source of the first absorption area (I), which is located about f = 1 Hz. It is more acceptable to use Maxwell–Wagner-Sillers polarization (MWS) in semi-crystalline polymers like PVC^27^. The existence of this phenomenon is thought to be caused by variations in the permittivities and conductivities of the components of the materials under investigation. The second absorption region (Π), located at about 10 Hz, could be attributed to the relaxation process affected by the interaction between the PVC main chain and plasticizer, added throughout sheet manufacturing, resulting in segments that are not significantly different from one another and have sizes smaller than those of PVC^27^. The third absorption zone (Ш) is attributed to the segmental motion of the polar groups that are attached to the main chain, with a relaxation duration of around 10^–4^ s^27^. It is seen that for the first process (I) (the low frequency process), the peak magnitude increases as the SSO concentration increases. This increase was related to the dipole density rising as the SSO concentration increased. The accumulation of impurities and free fatty acids in SSO at electrodes or interfaces may be the reason behind the rising dipole density^30^. Furthermore, Fig. 5 illustrates that, up to a concentration of 60/40, all relaxation processes shifted toward a lower frequency as the oil concentration increased. Bonding through dipole–dipole interactions between C=O groups in SSO and Cl atoms in PVC is expected to result in some compatibility between the two substances^31^. Plasticizers cause a reduction in intermolecular links between polymer chains (polymer–polymer interaction) by occupying space left by the polymer chains and replacing them with hydrogen bonds that are created by the interaction of the plasticizer with the polymer chains. By enabling more chain mobility, this rupture and reconstruction of polymer chain connections lower the stiffness and increase the ductile behavior of films^32^. The possible reason for the restricted behavior of PVC sheet chains is the domination of strong hydrogen bonds, Van Der Waals produced by polymer–polymer intermolecular interactions over PVC-SSO attraction, indicating less compatibility between PVC and SSO than that between PVC and the plasticizer added in PVC sheet first manufacturing. This perception is supported by an increase in PVC sheet/SSO composite crystallinity until 60/40, where the lower compatibility between PVC and SSO allowed polymer molecules to be rearranged in some orderly way. When a polymer crystallizes, the density of the crystalline phase increases, resulting in amorphous domain deformation. This deformation reduces the number of potential macromolecular conformations while increasing the relaxation time of "unfrozen" segmental mobility^32^. From Fig. 5, the relaxation time for the second process (Π) increased from about 0.02 to 0.3 s, while the relaxation time for the third process (Ш) increased from 3 × 10^–4^ to 2 × 10^–2^ s for 100/0 and 60/40, respectively. For the 50/50 blend, the behavior is reflected, and the relaxation processes shift toward high frequency again. A decrease in crystallinity may be the reason for the increase in PVC chain mobility after 60/40 film^33^. As shown later, these results agree with DSC, SEM, X-ray, and roughness results.

## DC conductivity

**Figure 6:**
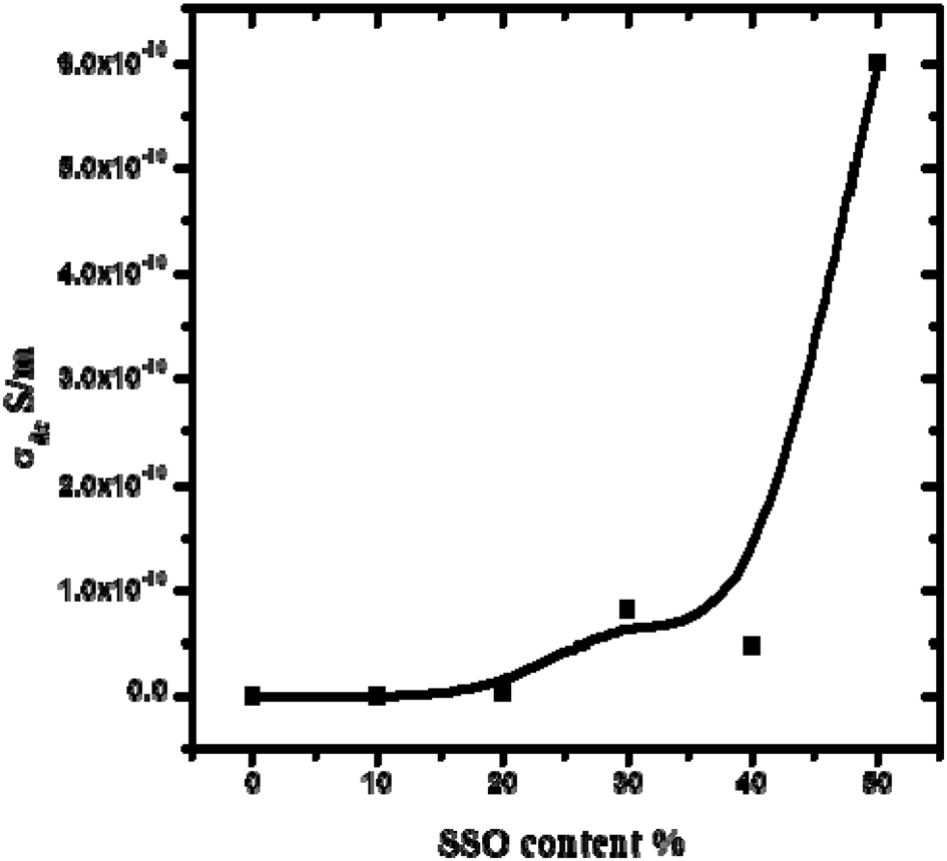
Dc conductivity σ_dc_ (S/m) of PVC sheet/SSO of ratios (100/0, 90/10, 80/20, 70/30, 60/40 and /5050) v/v% at 30 °C.

In a polymer system, dc conductivity is attributed to the space charge polarization at the blocking electrode. The obtained conductivity (from the conductivity term in the analysis curves) of blends as a function of SSO content at room temperature is shown in Fig. 6. From this figure, the conductivity slightly increases as the SSO content increases untill reaching 60/40. This increase is related to the charge density rising as the SSO concentration increased. The accumulation of impurities and free fatty acids at SSO interfaces may be the reason behind the rising charge density^31^. After 60/40 concentration, the conductivity increases significantly. This rapid rise could be due to the creation of SSO networks and a decrease in PVC sheet crystallinity^34^ The conductivity of the blends increases from about 5 × 10^–14^ to 6 × 10^–10^ for 100/0 and 50/50 PVC/SSO blends, respectively, which is located in the antistatic range^35^.

### Differential scanning calorimetry DSC

**Figure 7:**
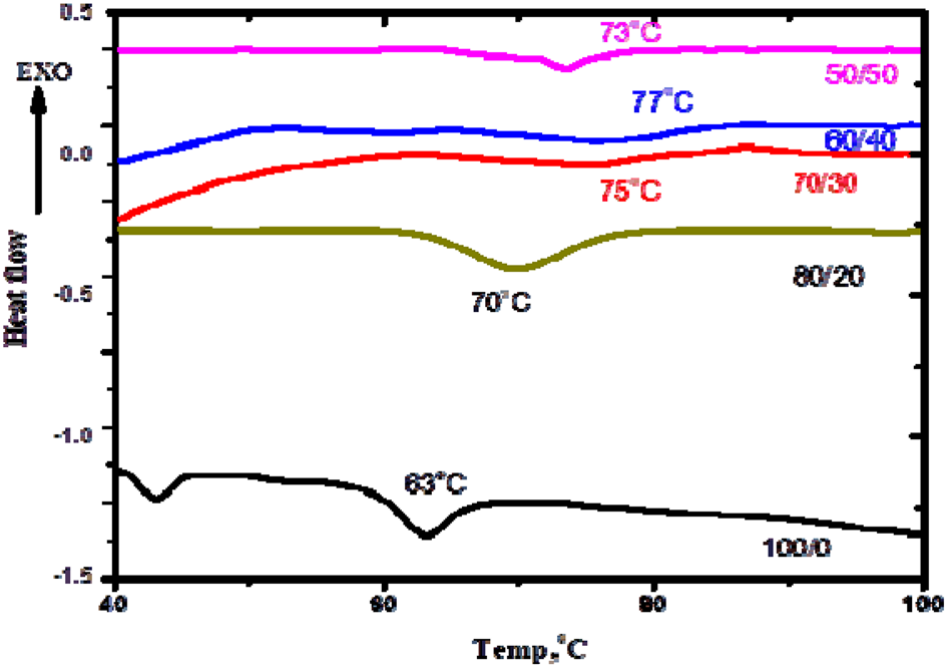
DSC heating thermogram of PVC sheet/SSO films of ratios (100/0, 80/20, 70/30, 60/40 and 50/50) v/v %.

The glass transition temperature (Tg) of all the sheets with various SSO concentrations was determined by using differential scanning calorimetry (DSC). The DSC curve is displayed in Fig. 7 Tg increased from about 63 to 77 °C for 100/0 and 60/40, respectively. Also, the DSC curve shows that the breadth of the glass transition grows as the SSO content increases until 60/40. After this concentration, for the 50/50 blend, the breadth and position of the glass transition decreased again ,Tg ~ 73 °C. This behavior at the glass transition temperature can be understood in terms of crystallinity. An increase in crystallinity reduces the mobility of the amorphous fraction at the crystal-amorphous interface, with amorphous chains closer to the crystal surface experiencing a greater degree of restriction. So The breadth and position of the glass transition temperature increased with increasing crystallinity^34,36^. This result is compatible with the X-ray result, which illustrates that the crystallinity of composites grows for 60/40 film, then decreases again for 50/50. However, all blend concentrations show some plasticization of PVC (Tg ranged from 63 to77 °C),where pure PVC has Tg above 80 °C. As a result, we can create more environmentally friendly PVC sheet/SSO composites.

### Thermogravimetric analysis TGA

**Figure 8:**
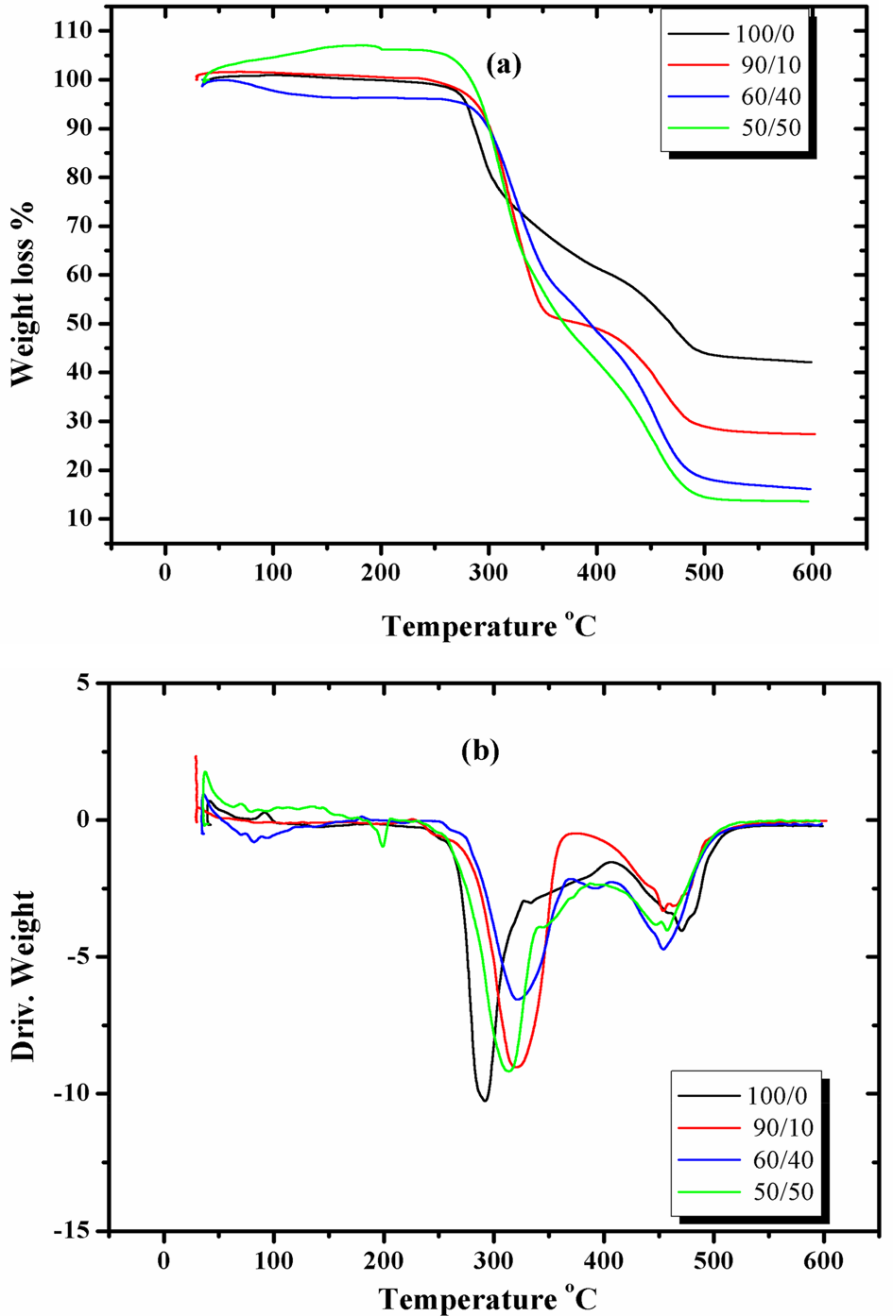
(**a**) TGA and (**b**) DTG curves for PVC sheet /SSO films of ratios (100/0, 90/10, 60/40 & 50/50) v/v%.

By thermogravimetric analysis, the mass loss of the measured samples was investigated while the temperature of the sample changed over time. The TGA and DTG curves of PVC sheet blended with SSO (100/0, 90/10, 60/40, and 50/50) v/v% are shown in Fig. 8a,b, and the obtained results are illustrated in Table 1. The thermal degradation of PVC sheet and its blends with SSO was investigated in the temperature range of 10 to 600 °C. As shown in Fig. 8a, in this range of temperatures, thermal degradation is thought to occur in two steps. Principally, the polymer is dehydrochlorinated in the first step, which occurs between 267.9 and 359 °C. This leads to the formation of conjugated double bonds, which break in the second step, which occurs between 550 °C and 785 °C. In the first step, HCl is the main volatile and produces low quantities of benzene and some other hydrocarbons. In the second step, the degradation of the dehydrochlorinated polymer continues with cracking and pyrolysis to form low-hydrocarbons of linear or cyclic structure^37^. It is clear from Graphs 8 (a and b) and Table (1) that the onset temperature T_o1_ of the first degradation step increases by SSO addition to the film up to 60/40, after which, at 50/50, it decreases again. This indicates that SSO increases the thermal stability of the films. This increase is due to the interaction between the evolved HCl ions and the oil, leading to the formation of short polyene sequences^27^. Furthermore, SSO could reduce the amount of evolved HCl, and T_o1_ will increase by increasing the amount of SSO in the film due to more interaction and a higher amount of produced polyenes. We also noticed that, at a blend ratio of 50/50, T_o1_ decreased, which means decreased thermal stability compared to other blends but still higher than PVC sheet without SSO. This may be due to the lower amount of HCl produced as the concentration of PVC decreased to 50%, and as a result, fewer polyenes will be produced. We also observe, in the second degradation step, that T_o2_ and the residual mass of the dehydrochlorinated polymer decreased with an increase in SSO and a decrease in PVC sheet in the blend. After studying the thermal analysis of the samples, we concluded that SSO could be used as a biostabilizer for PVC.

**Table 1:** Thermogravimetric data for PVC sheet /SSO films.

PVC sheet /SSO	Weight loss temperature °C						
	T_o1_	T_DTG1_	T_o2_	T_DTG2_	T_50%_	T_75%_	Residual mass (%)
100/0	267.98	291.4	446	470	468	0	44.6
90/10	288.9	319	433	453.9	382	0	27.5
60/40	286	320	431	454	392	467	19.2
50/50	276	312	426	457	369	455	13.5

### Optical properties

**Figure 9:**
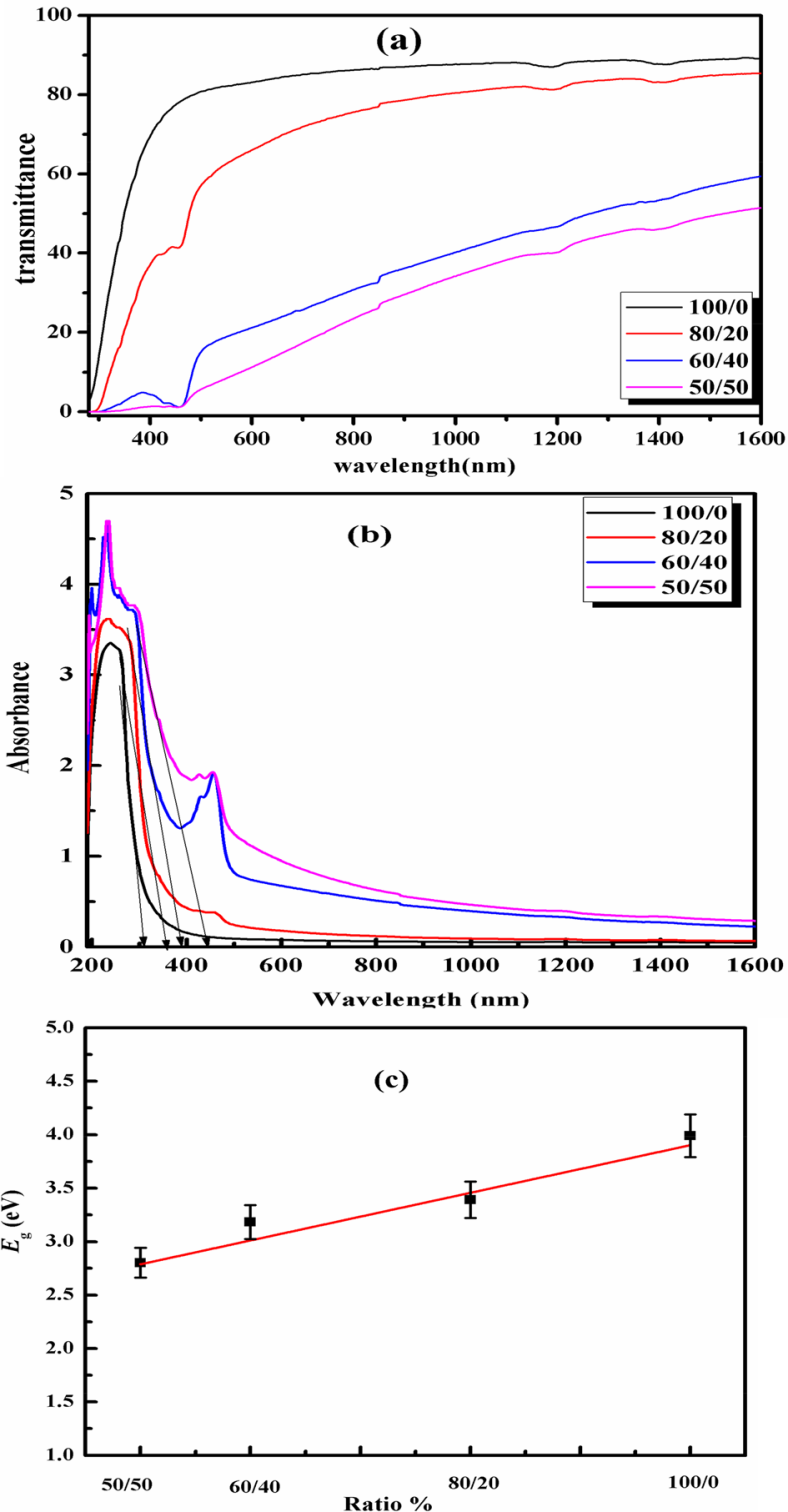
The UV/Vis. Transmittance % (**a**) and absorbance (**b**) spectra of PVC sheet/SSO films of ratios (100/0, 80/20, 60/40 and 50/50) v/v%.

In the UV-vis light region, the optical characteristics of PVC sheet/SSO films were investigated with regard to light transmittance; the findings are shown in Fig 9a. All the films had transmittance values of 0% in the UV range (≤350 nm). A complete blocking of ultraviolet (UV) light at wavelengths up to ∼350 nm may arise from the UV light absorption caused by the cross-linking components^38^. While the transmittance values in the UV range (≥350 nm) for PVC sheet range from 0 to 89% and for PVC sheet/SSO films of concentrations (80/20, 60/40, and 50/50) were from 0 to (85, 67, and 55%), respectively. The maximal transmittance (evaluated at the wavelength 1600 nm) of the film of ratio 50/50 was reduced by ∼34 percentage points compared to the PVC sheet film (i.e., from 89% to ∼55%). Also, incorporation of SSO increases the wavelength at which the film could transmit light. This indicates that the higher the SSO content, the higher the absorption capacity of the films in the visible light region, as shown in Fig 9b^21^. Both PVC sheet and its blends with SSO show strong absorption in the UV region. The PVC sheet /SSO films showed a greater absorption compared to the PVC sheet at wavelength λ = 238 nm. All the blended samples exhibit a shoulder-like band at 238 and 273 that may be attributed to the electronic transition π→π∗ (k band)^39^. As shown in Fig. 9b, the increase in absorption of all blended samples is evidence of the miscibility between the PVC sheet and the oil SSO. Furthermore, at higher wavelengths (λ = 424 and 454 nm), there is another absorption band appeared from the incorporation of SSO into the PVC sheet. This is attributed to the dehydrochlorenation and the interaction between the evolved HCL and SSO, leading to the formation of polyenes and the propagation of double bonds along the chain. Moreover, the number of released HCL decreased by SSO incorporation^40^. We also note that the sample with a ratio of 60/40 has the highest band intensity due to the highest number of formed polyenes, which decreased in 50/50 film. This means that the ratio 60/40 has the highest stability. From the foregoing, SSO acts as a stabilizer of the PVC sheet by capturing the Cl^−^ and H^+^ ions in the PVC chain. The energy band gap values Eg were calculated by using the equation in the experimental part and the values ellustrated in Fig. 9c. We note from the figure that the Eg values decreased as the content of SSO in the PVC sheet increased. It decreases by (15, 20, and 30%) for (80/20, 60/40, and 50/50) respectively.

### Surface roughness measurement

**Figure 10:**
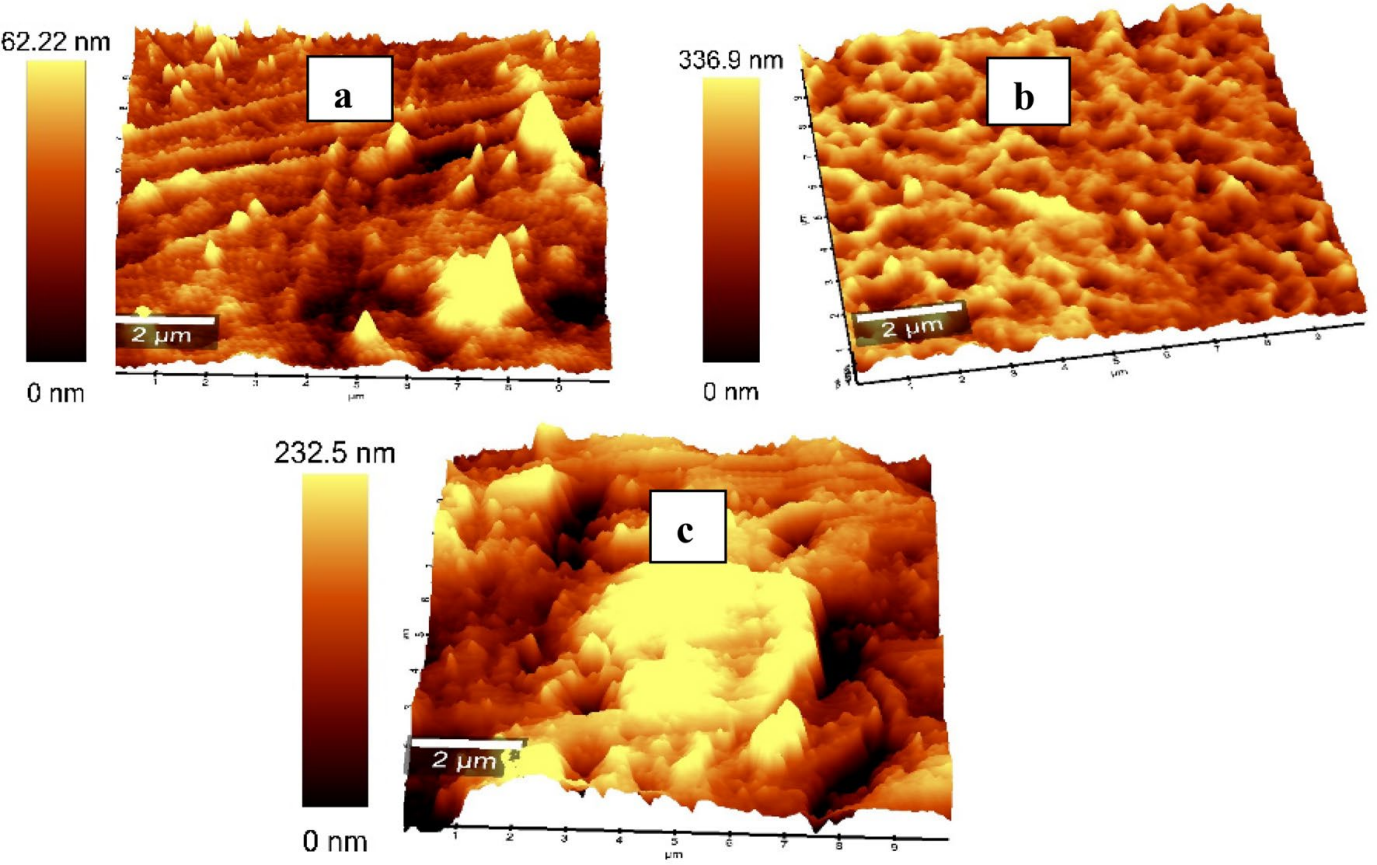
Atomic force microscope AFM for PVC sheet/SSO films of ratios (100/0, 60/40 and 50/50) v/v%.

The measurement of surface roughness is a very important technique for medical applications. Interfaces between different material layers often significantly affect the external properties of thin films. Surface roughness holds a commonality and critical impact among many material properties. In our study, the results obtained from AFM concerning, comparative analysis of the surface state of PVC sheet/SSO films (100/0, 60/40, and 50/50) v/v%, the results derived from the micrographs shown in Fig. 10a–c reveal that the surface topography for PVC sheet shows smooth and homogenous surface morphology, reflected by the small value of the maximum height of pores and the root mean square of roughness (62 and 77 nm). Instead, the incorporation of SSO led to an increase in the roughness of the PVC sheet/SSO bends, determined by an increase pore height, reaching 336.9 nm for the 60/40 blend ratio, then decreasing again as the concentration of SSO increased in the case of the 50/50 blend, which has a value of 232.5 nm. On the other hand, the root mean square of roughness increased as the concentration of SSO increased, reaching 88 and 114 nm for the 60/40 and 50/50 blend ratios, respectively, indicating a porous polymer structure of PVC sheet/SSO (60/40) due to the increase in pores number and decrease of their average diameters compared to (50/50), as seen in Fig. 10b,c, respectively^41^. We deduced from the results that the internal surface roughness increased with SSO addition due to SSO interference in the polymer matrix. Therefore, the blend latex is more likely to form a microphase separation state during the film formation process. The micro-phase separation leads to an increase in the surface roughness of the polymer film, therefore increasing the water and oil repellence of the polymer film surface, according to the Wenzel theory^13^. This enrichment of SSO at the surface and the rough surface morphology are keys to tailoring the polymer surface to exhibit excellent water and oil repellence at reduced total SSO content. At a concentration of 50/50, the film became supersaturated with SSO and became smooth due to oil accumulation, as seen in Fig. 10c, and the surface roughness decreased. This result is supported by dielectric results that showed a drop in the dielectric constant for the 60/40 PVC sheet/SSO blend.

### Contact angle measurement

**Figure 11:**
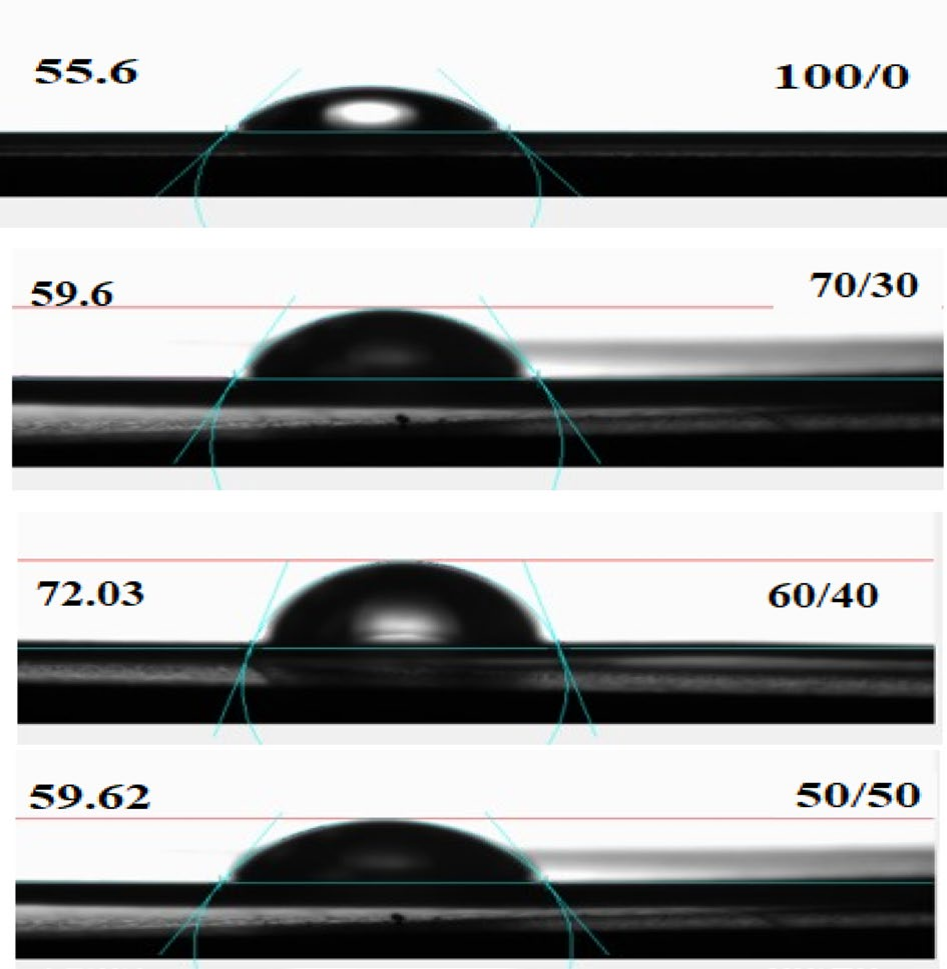
Contact angle of PVC sheet/SSO films of ratios (100/0, 70/30, 60/40 and 50/50) v/v%.

The hydrophilicity behavior of the films was investigated by water contact angle measurement. The mean contact angles of PVC sheet/SSO in ratios (70/30, 60/40, and 50/50) are summarized in Table 2 and their photos are shown in Fig. 11. The PVC sheet surface had a water contact angle of around 55.6^◦^, indicating its moderate hydrophilicity. The sheet surface is partially hydrophilic. After SSO incorporation, the mean contact angle was slightly increased to 59.6°, 72.03°, and 59.62° for the films of ratios (70/30, 60/40, and 50/50), respectively. The reason is probably that SSO enhances the hydrophobic nature and decreases the wettability of PVC membranes. The results also indicated that the degree of hydrophobicity (60/40) film significantly increased compared to the PVC sheet/SSO (100/0, 70/30, and 50/50) films. Our results indicated that the contact angle and surface roughness are correlated, meaning that as the contact angle increases, so does the surface roughness, which agrees with Ref.^42^. This is also comparable to Ref.^15^, who reported that according to the Wenzel equation, when the surface is hydrophobic, the contact angle increases as the roughness increases. Certain levels of roughness in hydrophobic materials allow for the maintenance of a space between solid-liquid surfaces.

**Table 2:** The contact angle of different PVC sheet/SSO films.

PVC sheet/SSO	100/0	70/30	60/40	50/50
Contact angle	55.6	59.6	72.03	59.62

### Hemocopatibility assay

The most important property of biomedical materials is their biocompatibility, especially their blood compatibility. Hemolysis rate is an important parameter for evaluating blood compatibility. It was reported that a value of up to 5% hemolysis is permissible for biomaterials^23^. The results of hemolysis%, clot weights, and clot index obtained for rat blood with PVC sheet/SSO films are summarized in Table 3. The optical densities of the positive control (100% lysis) and the negative control (0% lysis) were 2.8 and 0.008, respectively. The value of hemolysis % for PVC sheet/SSO film (100/0) was 1.96%. After SSO incorporation, the values of hemolysis% were 2.00, 2.46, 3.57, and 2.60% for the PVC sheet/SSO films of ratios (80/20, 70/30, 60/40, and 50/50) v/v %, respectively. There were slight differences in the value of hemolysis for PVC /SSO films compared to pure PVC sheet 100/0, as well as between PVC films with different SSO ratios. However, the values of the hemolysis% of all the films were located within the permissible limit with considerable biocompatibility^43^. The main reason for the reduced hemolysis rate is because RBCs were repelled by the negative charge of PVC when they come to the surface by electrostatic repulsion. Strong interactions between cells may be further inhibited, and hemolysis may be suppressed by the electrostatic repulsion. Also, the anti-thrombogenic potential of the surface of PVC sheet/SSO films was judged with a clot formation test. The different clot weights are related to the different abilities of the film surface to induce thrombus formation. The pure PVC sheet film (100/0) showed heavier blood clots than the other PVC sheet/SSO films, while the film with a ratio of 60/40 showed lighter blood clots than the other films. Additionally, the blood clotting index (BCI), which represents the free RBCs that are not trapped in clots, is used to express the clotting formation capacity. A lower BCI value means a higher clotting formation capacity. From the data, it was noticed that pure PVC sheet showed a lower BCI value than the other PVC sheet/SSO films, while the film of ratio 60/40 showed a higher BCI value than the other film ratios. Accordingly, PVC sheet/SSO (60/40) reduces blood clot formation and is considered non-hemolytic and biocompatible**.** This result is in accordance with^15^, who reported that surfaces with larger contact angles inhibit protein adsorption over longer periods of time than those with smaller contact angles by reducing contact between protein and the surface. Also, certain patterns, as well as roughness, can minimize surface contact and consequently reduce the available places for protein adsorption^44^. Also, one of the researchers explained that the rough surface is less thrombogenic than the smooth surface^45^.

**Table 3:** Blood compatibility parameters of different PVC sheet/SSO films.

PVC sheet/SSO	Clot weight(gm)	BCI		Hemolysis	
		A	%	A	%
100/0	0.231	0.175	20.3	0.063	1.96
80/20	0.2	0.224	26.1	0.064	2.00
70/30	0.185	0.37	43.1	0.077	2.46
60/40	0.167	0.57	66.4	0.108	3.57
50/50	0.207	0.285	33.2	0.081	2.60

## Conclusion

The incorporation of sunflower oil into PVC sheet with different ratios (100/0, 90/10, 80/20, 70/30, 60/40, and 50/50) greatly influences its dielectric, thermal, optical, and various surface properties such as roughness, contact angle, and blood compatibility. A straight correlation was found among these properties. SEM and FTIR measurements revealed good interference between the oil and the PVC sheet. Moreover, dielectric relaxation times confirmed the interaction between SSO and the PVC sheet which also supported by XRD results. DC conductivity increased to 6 × 10^–6^ S/m, so it could be applied in antistatic applications. Thermogravimetric analysis revealed that SSO increases the thermal stability of the PVC sheet by decreasing the hydrochlorination due to the interaction between the evolved HCl ions and the oil, leading to the formation of short polyene sequences, so SSO could be used as a biostabellizer and a bioplastizer for PVC. Studying surface roughness and contact angle indicated that as the surface roughness increased, the contact angle increased as well. These results were confirmed with hemolysis and blood clot measurements, which reveal that the ratio of 60/40 demonstrated a large decrease in thrombus weights along with a slight increase in hemolysis (%); all PVCS films' hemolysis% was, however, within the acceptable range and had a high degree of biocompatibility. From the foregoing, it is recommended to use SSO to be incorporated into PVC as a bioplasticizer, especially 60/40, to modify its biophysical properties to be used in blood bags as well as antistatic applications.

## Data Availability

All data generated or analyzed during this study are included in this published article.
